# Differential Role of Rapamycin and Torin/KU63794 in Inflammatory Response of 264.7 RAW Macrophages Stimulated by CA-MRSA

**DOI:** 10.1155/2014/560790

**Published:** 2014-04-01

**Authors:** Rebekah K. H. Shappley, Thomas Spentzas

**Affiliations:** ^1^Division of Pediatric Critical Care Medicine, University of Tennessee Health Science Center, Memphis, TN 38139, USA; ^2^Children's Foundation Research Institute at Le Bonheur Children's Hospital, 50 North Dunlap, Memphis, TN 38103, USA

## Abstract

*Background*. Rapamycin suppresses the RAW264.7 macrophage mediated inflammatory response but in lower doses induces it. In the present study, we tested the suppression of the inflammatory response in the presence of mTOR 1 and 2 inhibitors, Torin and KU63794. *Methods*. RAW264.7 cells were stimulated for 18 hrs with 10^6^ to 10^7^ CFU/mL inocula of community-acquired- (CA-) MRSA isolate, USA400 strain MW2, in the presence of Vancomycin. Then, in sequential experiments, we added Torin, KU63794, and Rapamycin alone and in various combinations. Supernatants were collected and assayed for TNF, IL-1, IL-6, INF, and NO. *Results*. Rapamycin induces 10–20% of the inflammatory cascade at dose of 0.1 ng/mL and suppresses it by 60% at dose of 10 ng/mL. The induction is abolished in the presence of Torin KU63794. Torin and KU63794 are consistently suppressing cytokine production 50–60%. *Conclusions*. There is a differential response between Rapamycin (mTOR-1 inhibitor) and Torin KU63794 (mTOR 1 and 2 inhibitors). Torin and KU63794 exhibit a dose related suppression. Rapamycin exhibits a significant induction-suppression biphasic response. Knowledge of such response may allow manipulation of the septic inflammatory cascade for clinical advantages.

## 1. Introduction

Sequential cytokine release from macrophages propels the inflammatory response in infection. An overwhelming cytokine release turns simple infection into sepsis [1, 2]. The host appears to mount a biphasic cytokine release response: first mounting a proinflammatory and then an anti-inflammatory response which quells the positive feedback loop of the cytokine storm [3]. The anti-inflammatory response or “immunoparalysis” refers to the diminished ability of the body to mount an appropriate cytokine storm and immune response and can lead to secondary infections [4, 5].

Our previous research has focused on the signaling pathways of the murine macrophage in response to CA-MRSA. We have shown that the agonism of the NMDA receptor increases and antagonism of the same receptor diminishes the TNF response [6]. Downstream from the NMDA, the phosphoinositide-3 kinase/akt (PI3/AKT) also influences such response [6, 7]. The next downstream signaling molecule is the mammalian target of Rapamycin (mTOR complex). The role of mTOR as a central controller of cell growth, proliferation, and metabolism has been described but many aspects are unclear [8, 9]. Previous studies by our group have shown that inhibition of mTOR by Rapamycin has a dose-dependent biphasic response on TNF secretion [7]. At very low doses, Rapamycin increases TNF production, but, at tenfold higher doses, TNF secretion is diminished. Rapamycin works by binding cytosolic FK-binding protein 12 (FKBP12) and together these form a complex that binds the mTOR complex 1 (mTORC1) [10–15]. mTORC1 is a serine/threonine phosphokinase which functions as a redox sensor, regulates cell growth and proliferation [16]. Rapamycin may act also on mTOR2 but it is not clear how this pathway influences the inflammatory cascade [17]. CD-34 derived dendritic cells undergo apoptosis when exposed to Rapamycin, but monocytes and macrophages do not [18].

It is known that these have various anabolic and catabolic activities; however, the relative role of mTORC1 and mTORC2 in macrophage cytokine stimulation is unknown.

There exist two mTORC1 and 2 inhibitors: KU63794 and Torin; although their role is not clear, it appears to exhibit more complete mTORC inhibition than Rapamycin [19, 20]. This study explores their role in inflammatory cascade in comparison to Rapamycin action. Investigation of the inflammatory pathway is important, since Rapamycin and Torin are currently in use in posttransplant and cancer patients. Additionally, knowledge of the pathway may also offer advantages in the immunomodulation of sepsis. In this paper, we hypothesize that the mTORC 1 and 2 inhibitors will have different effects on cytokine stimulation than Rapamycin in a murine macrophage model stimulated by CA-MRSA.

## 2. Methods

*Bacteria.* The bacteria selected for this study were the CA-MRSA strain MW-2, a Midwestern strain known to cause serious invasive disease. The MW-2 strain is representative of the USA-400 isolates, one of the major CA-MRSA pathogens in the United States [21]. Bacteria were grown at 37°C to the late logarithmic phase in tryptic soy broth (Becton, Dickinson and Company, Sparks, MD, USA), then washed in endotoxin-free phosphate-buffered saline. Colony counts were used to determine concentration of bacteria. Based on our previously published data with CA-MRSA we aimed for a concentration of bacteria of 10^6^-10^7^ cfu/mL [6, 7, 22]. The minimum inhibitory concentration was determined using the E-test method in the microbiology laboratory at Le Bonheur Children's Hospital (LBCH) and was susceptible to Vancomycin with MIC of <0.5 *μ*g/mL. 

*Cell Culture.* The cell culture consisted of RAW264.7 murine macrophage-like cells purchased from ATCC. The cells were cultured in Dulbecco's modified Eagle's medium (Mediatech, Herdon, VA, USA) and supplemented with 10% fetal bovine serum (HyClone, Logan, UT, USA) and 2 mM glutamine (GIBCO, Grand Island, NY, USA). For experiments, 1 × 10^6^ cells were placed in each well of 24-well tissue culture plates (Becton Dickinson, Franklin Lakes, NJ, USA).

Vancomycin was purchased through the Department of Pharmacy at LBCH from Hospira (Lake Forest, IL, USA) and is used to moderate the uncontrolled growth of MRSA and massive TNF production. A clinically achievable concentration of Vancomycin, 20 *μ*g/mL was added to the cell cultures prior to the addition of live staphylococci.

This was completed in parallel with various strengths of Torin (Tocris Bioscience, Bristol, UK) and KU63794 (Tocris Bioscience, Bristol, UK) to create a dose curve. These supernatants were harvested and analyzed (method below) and optimal dosing of Torin and KU63794 was identified. The tested concentrations of Vancomycin, Rapamycin, Torin, and KU63794 had no effect on the viability of the RAW264.7 cells as determined by visual inspection of the monolayer under a low power microscopic view.

Once optimal dosing of Torin and KU63794 were known, the experiment was repeated with combinations of Torin, Rapamycin (0.1, 1, 10, and 100 ng/mL) [18, 23], and KU63794. Control levels TNF, IL-1, IL-6, INF and NO (Rapamycin served as the control) were contrasted with stimulation in the presence of various combinations of mTORC1 inhibitor Rapamycin, and mTORC 1&2 inhibitor Torin, and mTORC 1&2 inhibitor KU63794.

After incubation, cell-free supernatants were collected and assayed for cytokine concentrations using solid phase sandwich enzyme-linked immunoabsorbent assay for TNF-*α* (eBioscience, San Diego, CA, USA), IFN (PBL Biomedical, Piscataway, NJ, USA), IL-1 (R&D Systems, Minneapolis, MN, USA), and IL-6 (eBioscience, San Diego, CA, USA). NO concentrations were examined using the Griess reaction (Promega, Madison, WI, USA). TNF secretion measurements were validated with an average of three well replicates performed three times, totaling nine samples. There exists intrinsic experimental variation within TNF, IL-1, IL-6, INF, and NO production in different cell culture flasks due to unique cell culture and endogenous macrophage differences, which is consistent with our previous studies [6, 7, 22, 24]. Cells from the same population were used to minimize variation for all experiments and the responses were ranked. 

*mTOR Activation.* The activation of mTOR was estimated by MSD phosphoprotein assay whole cell lysate kit. The fraction phospho(Ser2448)/total mTOR was computed at the 5 *μ*g cell lysate at 3 duplicates. The cells were exposed to MRSA without addition of mTOR inhibitors, and subsequently with the addition of Rapamycin 0.1, 1, 10, and 100 ng/mL, Torin 1 and 5 ng/mL, and KU63794 1 and 5 ng/mL. 

*Cells Viability*. KU63794, Torin, and Rapamycin at doses used in our experiments had no effect on the viability of the RAW264.7 cells as determined by a low-power microscopic inspection of the monolayer and exclusion of 0.2% trypan blue dye. Cell viability was confirmed using 3-(4,5-dimethylthiazol-2-yl)-5-(3-carboxymethoxyphenyl)-2-(4-sulfophenyl)-2H-tetrazolium, inner salt (MTS) according to the manufacturer's instructions (Promega, Madison, WI, USA) [25]. The MTS reagent is reduced by metabolically active cells into a colored formazan product whose absorbance is then measured. In brief, MTS solution was added to wells of a 96-well microtiter plate, and the cells were incubated for 2 h. The absorbance at 490 nm was then measured. 

*Data Analysis.* The data were analyzed with R 2.12.2 software. All the results are ranked and expressed as percent fold increase over the control. The percentile can easily be transformed to the actual value because the actual control value (pg/mL) for each experiment is given. For example, when the TNF response is 80% of the control and the actual control value is 33,561 pg/mL, then the response is 0.8 × 33,561 pg/mL = 26,849 pg/mL. The concentration of NO is measured in *μ*M. The data were graphed as boxplot mean and 1.96 SE.

The activation of mTOR computed as a fraction of the phospho- versus total mTOR and was graphed as boxplot.

## 3. Results

### 3.1. Torin and KU63794

**Figure 1:**
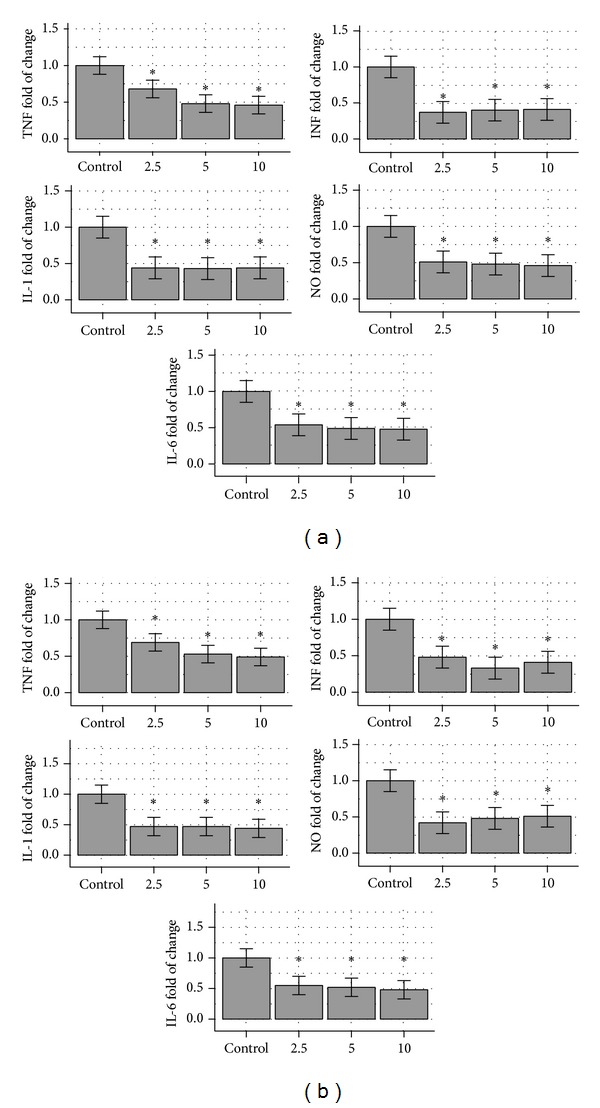
(a) A graphic representation of Table 1. The 1a indicates the reduction from the control of RAW264.7 macrophages inflammatory response of TNF, IL-1, IL-6, INF, and NO, respectively, in the presence of 0, 2.5, 5, and 10 ng/mL Torin. The graphs are depicted as a percent of the control response, that is, without Torin (0 ng/mL). TNF: tumor necrosis factor, IL-1: interleukin 1, IL-6: interleukin 6, INF: interferon, and NO: nitric oxide. (b) It is a graphic representation of Table 1. The 1b indicates the reduction from the control of RAW264.7 macrophages inflammatory response of TNF, IL-1, IL-6, INF, and NO, respectively, in the presence of 0, 2.5, 5, and 10 ng/mL KU63794. The graphs are depicted as a percent of the control response, that is, without KU63794 (0 ng/mL). TNF: tumor necrosis factor, IL-1: interleukin 1, IL-6: interleukin 6, INF: interferon, and NO: nitric oxide.

First, the effects of 0, 2.5, 5, and 10 pg/mL doses of Torin and KU63794 were tested. Without Torin, the MW2 CA-MRSA stimulated RAW264.7 macrophages produced 33,561 pg/mL TNF and this production was used as control, that is, 100% response. The addition of 2.5 ng/mL reduced the response to 22,821 pg/mL or 68% (*P* < 0.05). Further increase of added Torin at 5 and 10 ng/mL reduced the TNF to 16,109 pg/mL (48%) and 15,438 pg/mL (46%). Those reductions were different from the control (*P* < 0.05) and the 2.5 pg/mL of Torin but not statistically different from each other. The effects on other cytokines also show reduction from the control. IL-1 was produced at 555 pg/mL without Torin (control 100%) and was reduced to 244 pg/mL (44%), 239 pg/mL (43%), and 245 pg/mL (44%) at doses of 2.5, 5, and 10 pg/mL, respectively. Although all were different than the control (*P* < 0.05), they were not statistically different from each other. Therefore, the dose effect reduction of 2.5 versus 5 or 10 ng/mL of Torin observed with TNF was not seen with IL-1. IL-6, INF, and NO produced similar pattern reduction as seen with IL-1. The Torin effects on TNF, IL-1, IL-6, INF, and NO are presented in Table 1 (upper part) and depicted as graphs in Figure 1(a).

The responses with KU63794 were similar. Without KU63794, the MW2 CA-MRSA stimulated RAW264.7 macrophages produced and average of 32,865 pg/mL TNF (slightly different than the average 33,561 pg/mL observed in the Torin series), and this average was used as a control, that is, a 100% response. Addition of KU63794 at 2.5, 5, and 10 ng/mL reduced the production to 22,677 pg/mL (69%), 17,417 pg/mL (53%), and 16.104 pg/mL (49%). All these values are different than the control (*P* < 0.05) and the response at 2.5 ng/mL is different than the response at 5 ng/mL or 10 ng/mL (*P* < 0.05). IL-1 was produced at 432 pg/mL without Torin (control 100%) and was reduced to 202 pg/mL (47%), 203 pg/mL (47%), and 190 pg/mL (44%) at 2.5 pg/mL, 5 pg/mL, and 10 pg/mL, respectively. All KU63794 additions are different than the control (*P* < 0.05) but not from each other; that is, the dose effect is not present. The KU63794 effects on TNF, IL-1, IL-6, INF, and NO are presented in Table 1 (lower part) and depicted as graphs in Figure 1(b). Our dose curves experiments indicated that Torin and KU63794 have diminished suppression (~80–99% of the control) at doses of 2, 1.5, and 1 ng/mL and plateau to not statistically significant different values at doses 0.9, 0.7, 0.5, 0.3, 0.1, and 0.01 ng/mL.

The viability of the cells was tested as described in Section 2. There were no changes in the spectrum of tested doses.

### 3.2. Rapamycin and Torin

**Figure 2:**
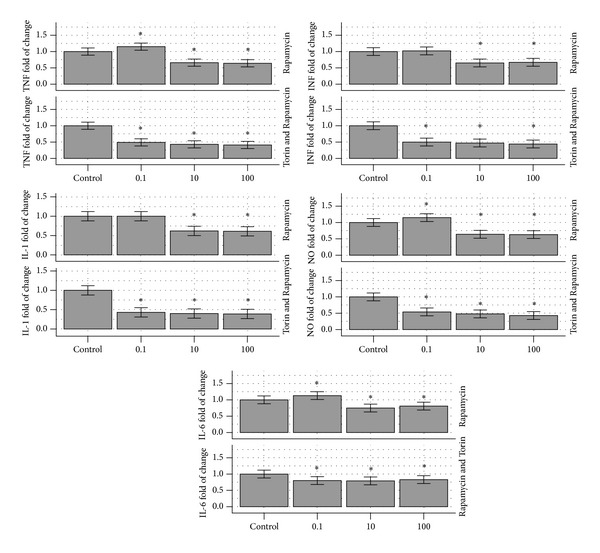
A graphic representation of Table 2. It indicates the reduction from the control of RAW264.7 macrophages inflammatory response of TNF, IL-1, IL-6, INF, and NO, respectively, in the presence of 0, 0.1, 10, and 100 ng/mL Rapamycin (up) and Rapamycin and Torin 5 ng/mL (down). The graphs are depicted as a percent of the control response, that is, without Rapamycin (up) or Rapamycin and Torin (down). TNF: tumor necrosis factor, IL-1: interleukin 1, IL-6: interleukin 6, INF: interferon, and NO: nitric oxide.

The effects of 0, 0.1, 10, and 100 ng/mL Rapamycin were compared to the same Rapamycin dosing and Torin 5 ng/mL. The stimulated macrophages produced an average of 32,758 pg/mL of TNF without Rapamycin and this was used as control (100%). When Rapamycin was added at doses of 0.1, 10, or 100 ng/mL, the average production of TNF was 37,672 pg/mL (115%), 21,620 pg/mL (66%), and 20,965 pg/mL (64%), respectively. All the responses are different than the control (*P* < 0.05). However, the 0.1 ng/mL dose response is different than the 10 ng/mL and 100 ng/mL and increases rather than suppresses the TNF response. The IL-1 response without Rapamycin was 486 pg/mL (establishing the 100% control) and similar was the response with the addition of Rapamycin 0.1 ng/mL (486 pg/mL: 100%). However, at 10 and 100 ng/mL Rapamycin, the IL-1 response decreased to 301 ng/mL (62%) and 297 ng/mL (61%); both differed significantly from the control. When Torin 5 ng/mL was added to the same experiment (Rapamycin at doses 0, 0.1, 10, and 10 ng/mL), a reduction of 49%, 43%, and 41%, respectively, was noticed. All were significant different than the control response of 34,231 pg/mL (*P* < 0.05) but not significantly different than each other. Therefore, the increase of cytokine response observed at 0.1 ng/mL of Rapamycin alone was eliminated (100% response without Torin and 49% with Torin). Similar results were noticed with the other cytokines. The results of the other cytokines are presented in Table 2 and Figure 2. The viability of the cells during the experiment was tested as described in Section 2.

### 3.3. Rapamycin and KU63794 5 ng/mL

**Figure 3:**
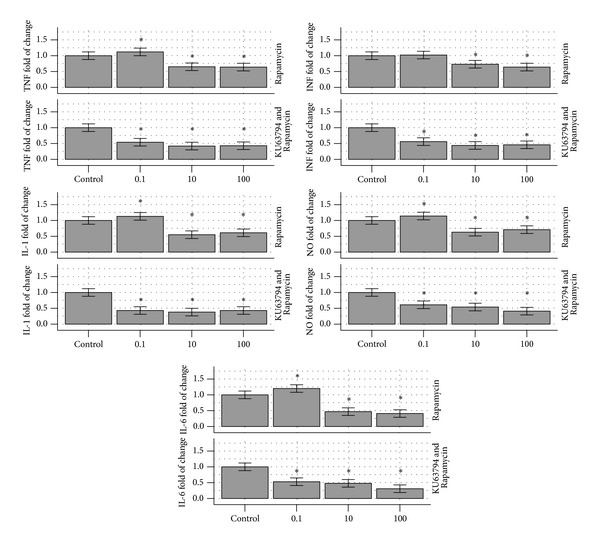
A graphic representation of Table 3. It indicates the reduction from the control of RAW264.7 macrophages inflammatory response of TNF, IL-1, IL-6, INF, and NO, respectively, in the presence of 0, 0.1, 10, and 100 ng/mL Rapamycin (up) and Rapamycin and KU63794 5 ng/mL (down). The graphs are depicted as a percent of the control response, that is, without Rapamycin (up) or Rapamycin and KU63794 (down). TNF: tumor necrosis factor, IL-1: interleukin 1, IL-6: interleukin 6, INF: interferon, and NO: nitric oxide.

Similar to the Rapamycin-Torin experiment, the effects of 0, 0.1, 10, and 100 ng/mL of Rapamycin were compared to the same Rapamycin dosing and KU63794 5 ng/mL. The control without Rapamycin was 32,865 pg/mL and addition of 0.1 ng/mL Rapamycin increased the TNF average to 42,171 pg/mL, that is, 120%, as was observed in the previous experiment. The 10 and 100 ng/mL of Rapamycin addition decreased TNF to 65% and 64% to the control level, all of which were significantly different (*P* < 0.05). KU63794 at 5 ng/mL blunted the 0.1 ng/mL Rapamycin-induced TNF response to 69% of the control. The 10 and 100 ng/mL of Rapamycin with 5 ng/mL KU63794 were 53% and 49%, all significantly different than the control (0.05), but not from each other. The IL-1 response had a similar pattern with an increase at 0.1 ng/mL of Rapamycin (113% of the control) but a decrease to 43% of the control, when KU63794 5 ng/mL was added. The results are presented in Table 3 and depicted as graphs in Figure 3. The viability of the cells was tested as described in Section 2.

### 3.4. Activation of mTOR

The phosphorylated/total mTOR cell fraction for the control (stimulated by MRSA cells without inhibitor) was 1.82. At the presence of Rapamycin 0.1, 1, 10, and 100 ng/mL, the results were 1.56, 0.82, 0.46, and 0.43; thus, increasing dose of Rapamycin exhibited incremental suppression.

**Figure 4:**
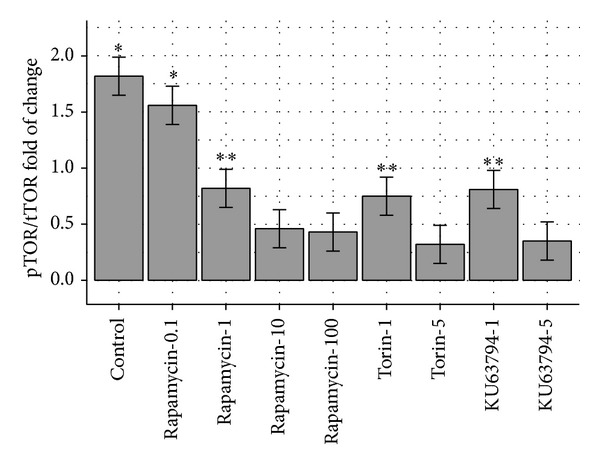
A graphic representation of activated mTOR expressed as phosphor/total mTOR. It indicates the activated fraction at the control (without inhibitor) with Rapamycin at doses 0.1, 10, and 100 ng/mL, Torin at doses 1 and 5 ng/mL, and KU63794 at doses 1 and 5 ng/mL. Rap-0.1: Rapamycin 0.1 ng/mL, Rap-1: Rapamycin 1 ng/mL, Rap-10: Rapamycin 10 ng/mL, Rap-100: Rapamycin 100 ng/mL, Tor-1: Torin 1 ng/mL, Tor-5: Torin 5 ng/mL, KU-1: KU63794 1 ng/mL, and KU-5: KU63794 5 ng/mL.

When Torin or KU63794 at dose 1 or dose 5 ng/mL was added, the fraction was 0.75, 0.32, 0.81, and 0.35 respectively. Therefore, the phosphorylated/total mTOR fraction at the presence of inhibitors, Rapamycin, Torin, or KU63794, was dose-dependent in all inhibitors. The activation fraction was the same for Rapamycin 10 or 100 ng/mL, Torin 5 ng/mL, or KU63794 5 ng/mL used (*P* < 0.05)—see Figure 4.

## 4. Discussion

The roles of Rapamycin, an incomplete dose-dependent mTORC inhibitor, and the more potent mTOR inhibitors Torin/KU63794 are explored in the context of severe inflammation.

Consistent with our previous studies, we found that low-dose Rapamycin does not suppress but instead stimulates TNF production [7]. At higher doses, Rapamycin suppresses production of these cytokines. However, when combined with Torin or KU63794, we find that the induction effect is no longer present. Higher dose of Rapamycin alone or with Torin or KU63794 suppresses cytokine production. The data shows this suppression to be consistent with the production of TNF, INF, IL-1, IL-6, and NO. Although the Rapamycin 0.1 ng/mL induction was relatively small (10–20%), it has significantly different response if it is compared with the 60% suppression induced with higher doses. Such difference (60%) may allow significant cytokine and clinical response manipulation at various stages of sepsis. It can possibly explain the variability in clinical course occasionally seen in septic patient taking Rapamycin. Torin and KU63794 suppress the TNF, INF, IL-1, IL-6, and NO up to 50% of the control in a dose related mode without exhibiting an induction.

The assessment of phospho(Ser2448)/total mTOR at various Rapamycin doses was not able to explain the biphasic TNF elevation, since it was dose-dependent as expected. Similarly, Torin and KU63794 exhibited also dose-dependent suppression.

Rapamycin has been described as dose-dependent inhibitor in cancer cell metabolism [26–28]. The more complete inhibition of Torin and KU63794 has been generally attributed to mTORC2 inhibition, although more complete inhibition of mTORC1 is possible [19, 29]. Rapamycin's mTORC inhibition is also dependent on phospholipase D activity. Elevated phospholipase D seems to increase the Rapamycin resistance to mTOR inhibition [26, 27, 30, 31].

We surmise that the suppression of cytokine production is a result of the relative amount of inhibition of mTORC1 and mTORC2, since phospholipase D activity had to be similar between the experiments. Thus, the proportional inhibition of mTORC2 or more complete inhibition of mTORC1 appears to have a suppressive effect on cytokine production in a murine macrophage model.

There is an inherent limitation of applicability when comparing in vitro murine macrophages to an in vivo model. The influence of Rapamycin to the Staphylococcal Toxic Shock has been studied [32, 33]. However, our findings add the existence of a Rapamycin dose diphasic effect in the cytokine production. Such effect does not exist in other mTOR inhibitors like Torin and KU63794.

## 5. Conclusion

Rapamycin offers a significant biphasic induction and suppression of the inflammatory cascade. Torin and KU63794 offer a dose related suppression of inflammatory cytokines. Addition of Torin and KU63794 appears to blunt the Rapamycin induction and converting it to a dose related suppression. The present study performed in a cell culture sepsis simulation model describes the difference but more studies are needed to define the exact contribution of mTOR1 and mTOR2. However, understanding and further exploring the differential response of mTOR inhibitors in inflammation can lead to a clinically advantageous cytokine modulation based on Rapamycin dosing or Torin and KU63794 combination. The battle of sepsis especially in immunosuppressed patients requires understanding of the balance of immune overactivity and immunosuppression. Small but decisive regulations at appropriate phases may offer significant clinical advantages.

## Figures and Tables

**Table 1:** The table indicates the reduction from the control of RAW264.7 macrophages inflammatory response in the presence of 0, 2.5, 5, and 10 ng/mL Torin (up) and KU63794 (down).

	TNF (%-pg/mL)	IL-1 (%-pg/mL)	IL-6 (%-pg/mL)	INF (%-pg/mL)	NO (%-*μ*M)
Torin (ng/mL)					
0	100% 33,561 ± 4,027	100% 555 ± 83	100% 845 ± 127	100% 96 ± 14	100% 25.3 ± 3.8
2.5	68%*	44%*	54%*	37%*	51%*
5	48%*	43%*	49%*	40%*	48%*
10	46%*	44%*	48%*	41%*	46%*
KU63794 (ng/mL)					
0	100% 32,865 ± 3,615	100% 432 ± 52	100% 743 ± 89	100% 95 ± 11	100% 23.2 ± 2.8
2.5	69%*	47%*	55%*	48%*	42%*
5	53%*	47%*	52%*	33%*	48%*
10	49%*	44%*	48%*	41%*	51%*

The first line indicates the response without Torin or KU63794 (0 ng/mL) which is used as control (100% response) and the subsequent values are expressed as % of such responses.

*Indicates statistical significance (*P* < 0.05) from the control.

TNF: tumor necrosis factor, IL-1: interleukin 1, IL-6: interleukin 6, INF: interferon, and NO: nitric oxide.

**Table 2:** The table indicates the reduction from the control of RAW264.7 macrophages inflammatory response in the presence of 0, 0.1, 10, and 100 ng/mL Rapamycin (up) and Rapamycin and Torin 5 ng/mL (down).

	TNF (%-pg/mL)	IL-1 (%-pg/mL)	IL-6 (%-pg/mL)	INF (%-pg/mL)	NO (%-*μ*M)
Rapamycin (ng/mL)					
0	100% 32,758 ± 3,603	100% 486 ± 58	100% 799 ± 96	100% 87 ± 10	100% 22.1 ± 2.7
0.1	115%*	100%	113%*	102%	115%*
10	66%*	62%*	75%*	65%*	64%*
100	64%*	61%*	81%*	67%*	63%*
Rapamycin and Torin at 5 ng/mL					
0 and no Torin	100% 34,231 ± 3,765	100% 396 ± 48	100% 831 ± 100	100% 89 ± 11	100% 22.7 ± 2.7
0.1	49%*	43%*	80%*	50%*	54%*
10	43%*	40%*	79%*	47%*	48%*
100	41%*	39%*	83%*	44%*	43%*

The first line indicates the response without Rapamycin (up) or without Rapamycin and Torin (down) which is used as control (100% response) and the subsequent values are expressed as % of such responses.

*Indicates statistical significance (*P* < 0.05) from the control.

TNF: tumor necrosis factor, IL-1: interleukin 1, IL-6: interleukin 6, INF: interferon, and NO: nitric oxide.

**Table 3:** The table indicates the reduction from the control of RAW264.7 macrophages inflammatory response in the presence of 0, 0.1, 10, and 100 ng/mL Rapamycin (up) and Rapamycin and KU63794 5 ng/mL (down).

	TNF (%-pg/mL)	IL-1 (%-pg/mL)	IL-6 (%-pg/mL)	INF (%-pg/mL)	NO (%-*μ*M)
Rapamycin (ng/mL)					
0	100% 35,143 ± 4,217	100% 496 ± 60	100% 834 ± 100	100% 91 ± 11	100% 24.3 ± 2.9
0.1	120%*	113%*	120%*	102%	110%*
10	65%*	55%*	47%*	73%*	63%*
100	64%*	61%*	41%*	64%*	71%*
Rapamycin and KU63794 at 5 ng/mL					
0	100% 32,865 ± 3,944	100% 432 ± 42	100% 743 ± 89	100% 95 ± 11	100% 23.2 ± 2.8
0.1	69%*	43%*	53%*	56%*	61%*
10	53%*	38%*	48%*	44%*	54%*
100	49%*	43%*	31%*	46%*	41%*

The first line indicates the response without Rapamycin (up) or without Rapamycin and KU63794 (down) which is used as control (100% response) and the subsequent values are expressed as % of such responses.

*Indicates statistical significance (*P* < 0.05) from the control.

TNF: tumor necrosis factor, IL-1: interleukin 1, IL-6: interleukin 6, INF: interferon, and NO: nitric oxide.

## Highlights

(i) Rapamycin exhibits a dose related biphasic influence to the inflammatory cascade. (ii) Torin and KU63794 (mTOR 1 and 2 inhibitors) suppress the inflammatory response. (iii) Addition of Torin or KU63794 converts Rapamycin's response to suppression.
